# Conservation implications of asymmetric introgression and reproductive barriers in a rare primrose species

**DOI:** 10.1186/s12870-019-1881-0

**Published:** 2019-06-28

**Authors:** Yongpeng Ma, Tobias Marczewski, Dan Xue, Zhikun Wu, Rongli Liao, Weibang Sun, Jane Marczewski

**Affiliations:** 1 Yunnan Key Laboratory for Integrative Conservation of Plant Species with Extremely Small Populations, Kunming Institute of Botany, Kunming, 650201 Yunnan China; 2 Yunnan Agricultural University, Kunming, 650201 Yunnan China

**Keywords:** Asymmetric introgression, Conservation implication, Genetic swamping, Heterostyly, Reproductive isolation

## Abstract

**Background:**

*Primula* is a large genus of flowering herbs well known for their heterostyly. Currently few natural hybrids are known and reproductive barriers in this genus in the wild have received little attention. However, there is instance of hybridization between rare and widely-spread species, and conservation implications of such situation is poorly understood. In the present study, we investigated hybridization patterns and reproductive barriers between a wide spread species, *Primula poissonii* and a rare species *P. anisodora*, of which only three populations are currently known.

**Results:**

Pollinator-mediated reproductive isolation was strong between parental species but not significant between hybrids and parental species. Hand pollination experiments showed significant reduction of both fruit- and seed-set for heterospecific pollination as compared with conspecific pollination for both parental species. Furthermore, hybrids had higher fruit- and seed-set when pollinated with *P. anisodora* pollen as opposed to *P. poissonii* pollen. Microsatellites identified backcrosses to *P. anisodora* in two of the three populations of *P. anisodora,* and additionally more individuals of *P. anisodora* showed introgression from *P. poissonii* than vice versa.

**Conclusions:**

These results provide evidence for potential genetic swamping of the *P. anisodora* populations, which could pose a serious threat for this locally endemic species.

**Electronic supplementary material:**

The online version of this article (10.1186/s12870-019-1881-0) contains supplementary material, which is available to authorized users.

## Background

Natural hybridization is of significance in the study of several aspects of evolution, including the origins of new ecotypes or species, the origin and transfer of genetic adaptations, and the reinforcement or breakdown of reproductive barriers [1–3]. However, natural hybridization can also be a serious threat to rare plant species because of pollen and/or ovule discounting in the process of hybridization, if hybrids are sterile and/or have low viability. Furthermore, there can be a risk of genetic swamping by which partially fertile and viable hybrids are replacing pure parental genotypes [4, 5].

It is generally believed that habitat disturbance can be a driving force for hybridization [6–8], as it can alter patterns of contact between reproductively compatible species, favoring successful establishment of hybrids due to, for example, the creation of suitable intermediate habitats. Human mediated hybridization is expected to increase worldwide and therefore potential threats to rare species are becoming more serious than ever before. In developed countries (e.g. US, Canada) conservation issues arising from hybridization have been well explored and even addressed by national conservation regulations [4, 7, 9, 10]. In contrast, there seems to be little research effort in developing countries to address the matter, which impacts negatively on the awareness of policymakers with regards to the involved threats posed by anthropogenic change of the environment.

Though hybridization in plants has been frequently reported, most cases involve wide spread species with limited numbers of hybrid zones occurring in their sympatric distribution areas [7, 11–13]. In such scenarios, there is little risk to either species, as geographical isolation is still strong enough to maintain populations of both species with minimal to no introgression. If, however, one of the hybridizing species has a narrow distribution, all of its populations might be exposed to geneflow from the other species.

*Primula* is a large plant genus (> 500 species) that is well known for its heterostyly. Interspecific hybridization and reproductive isolation have been studied for over a century in two European species; however other species of the genus have received barely any attention in this regard [13]. In the present study we investigated patterns of hybridization and reproductive barriers between the wide spread species *Primula poissonii* and the rare species *P. anisodora*, for which only three populations are currently known. Both of the species are heterostylous. Our aims were to (1) clarify the genetic structure of a hybrid swarm between these two species, and (2) investigate how reproductive barriers contribute to the pattern of hybridization; and then (3) assess the role of hybridization for the conservation of *P. anisodora* in order to develop meaningful conservation management strategies.

## Results

### Pollinator mediated reproductive isolation (RI)

For the pollinator observations in the natural setting, focusing on pure parental species, we observed a total of 804 visits by insects to flowers of *P. poissonii*: 347 visits by bumblebees (43%), 224 by bees (*Anthophora* sp., 28%), 217 by butterflies (27%) and 16 by hoverflies (2%). For *P. anisodora* plants, 435 visits were recorded: 336 by bumblebees (77%), 96 by bees (*Anthophora* sp., 22%) and only three visits by butterflies (0.3%). Although bumblebees are the dominant pollinator for both parental species, they make up a significantly higher proportion of overall visits for *P. anisodora* (77% vs 43%, χ^2^ = 131.2; *p* < 0.001). Furthermore, butterflies were important pollinators for *P. poissonii*, but seemed to only accidentally end up on a *P. anisodora* flower.

A total of 124 pollination bouts were observed in Plot 1, including 31 cross-specific and 93 con-specific bouts. A total of 276 transitions were observed, including 71 heterospecific transitions. Thus the overall ethological RI in Plot 1 between parental species = 1–31/124 * 71/276 = 0.940. For Plot 2, a total of 144 bouts were observed, including 39 cross-specific and 105 con-specific bouts. A total of 443 transitions were observed, including 88 bouts with heterospecific transitions. The overall ethological RI in Plot 2 between parental species = 1–39/144 * 88/443 = 0.946.

**Fig. 1:**
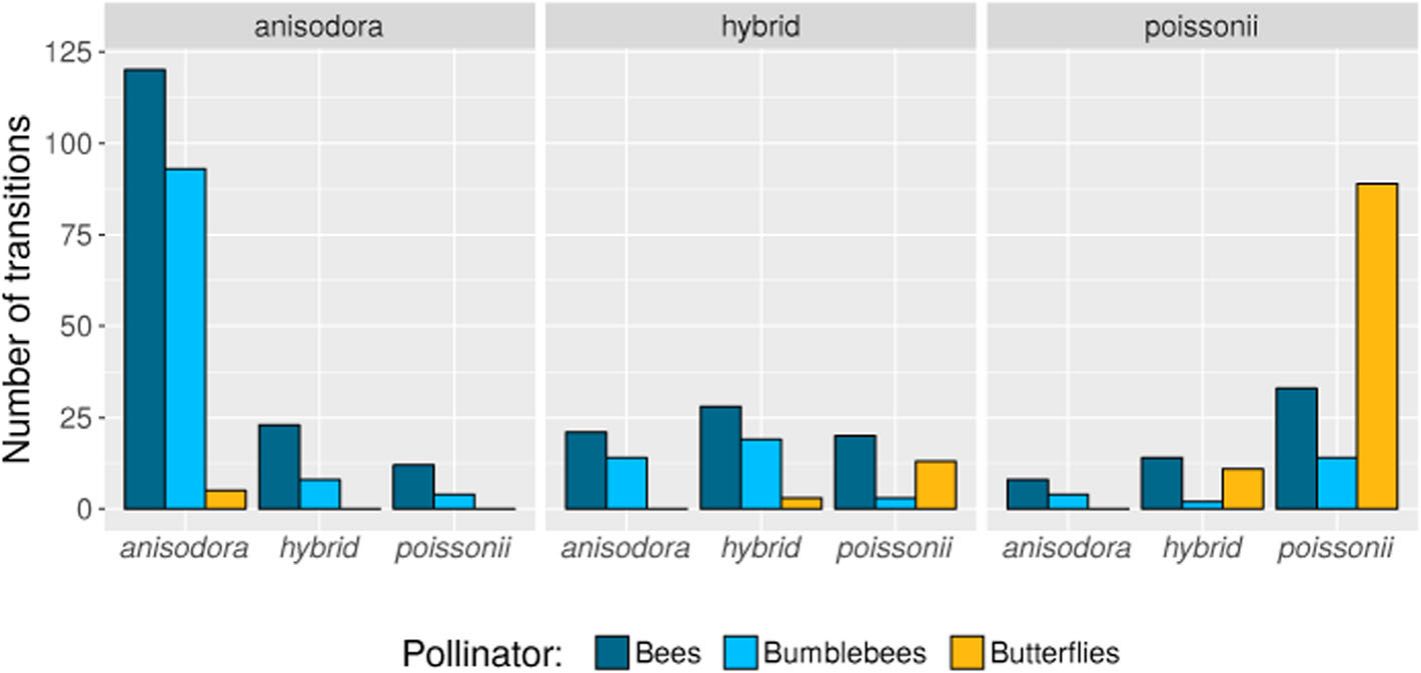
Number of transitions between *P. poissonii*, hybrids and *P. anisodora* within bouts of three pollinator groups. On the top the currently visited species is indicated, and on the x-axis which species the pollinator had visited immediately before. Pollinators were: Bees (*Anthophora sp.*), Bumblebees and Butterflies (*Aporia bieti*). Most visits of butterflies were restricted to *P. poissonii* with some occasionally visiting hybrids, while bees and bumblebees mostly visited *P. anisodora*, but were less selective, with several visiting hybrid and *P. poissonii* flowers

**Fig. 2:**
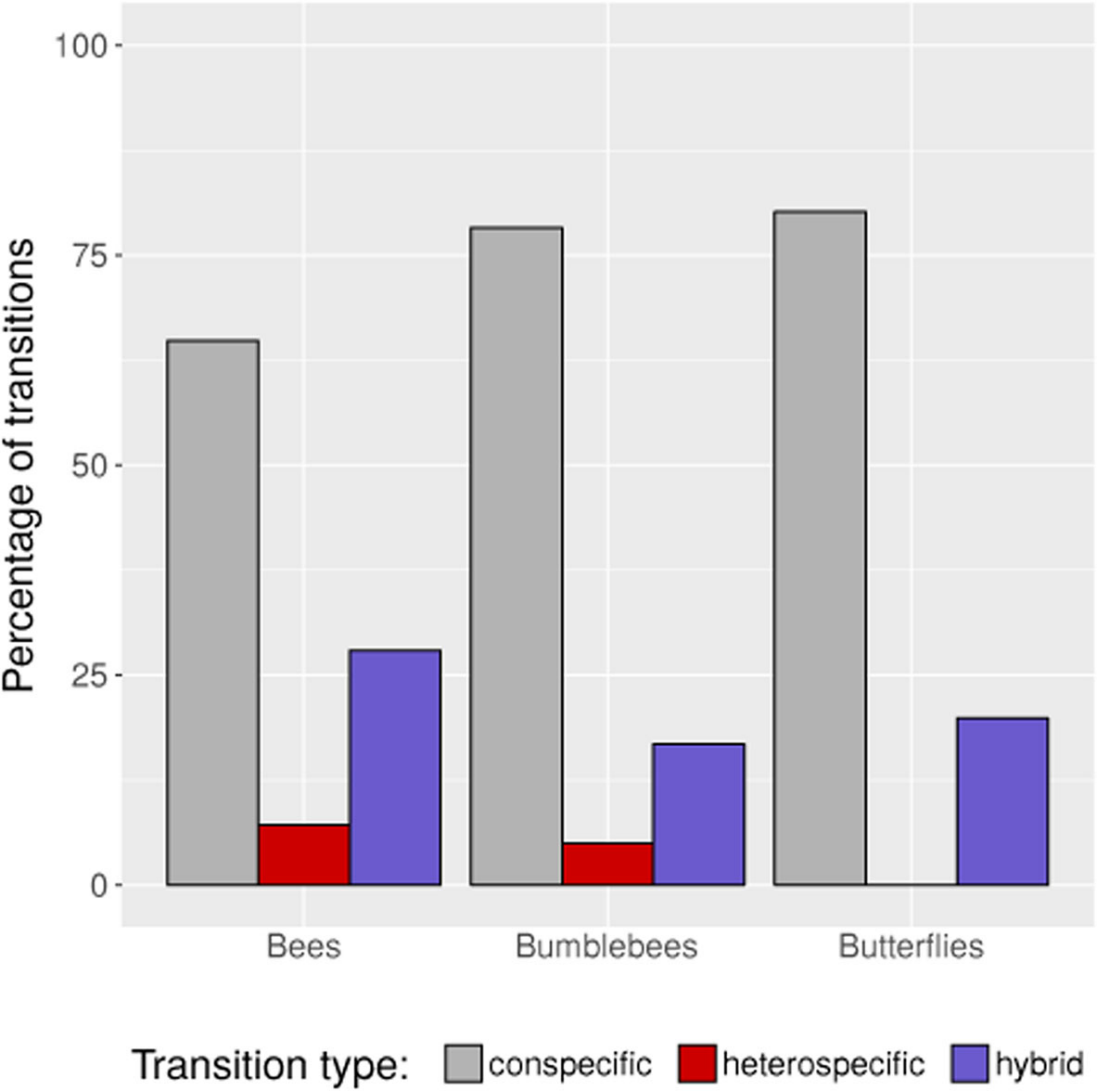
Transition percentages for three groups of pollinators: Bees (*Anthophora sp.*), Bumblebees and Butterflies (*Aporia bieti*) observed in bouts involving *P. anisodora*, *P. poissonii* and hybrids. Transition types were categorised as: conspecific (anisodora-anisodora, poissonii-poissonii, hybrid-hybrid), heterospecific (anisodora-poissonii, poissonii-anisodora), or hybrid (anisodora-hybrid, poissonii-hybrid, hybrid-anisodora, hybrid-poissonii). Most transitions were conspecific for all pollinator groups, and heterospecific transitions were relatively rare. Hybrids were much more often included in non-conspecific bouts than the respective other species, suggesting weakening of pollinator-mediated isolation when hybrids are present

In Plot 3, 114 bouts (39 solely within species/hybrids) were observed, and a total of 561 transitions were observed from one plant to another. Of these transitions, 404, accounting for 72% of the total, were within taxa. The remaining 157 transitions, accounting for 18% of the total, occurred between species (Fig. 1; Additional file 3: Table S3). The group of pollinators for which most visitations, and hence transitions, was observed were bees (*Anthophora sp.*), but for *P. poissonii* butterflies (*Aporia bieti*) were much more frequent visitors (Fig. 1). For butterflies no heterospecific transition was observed between the two species, but transitions between *P. poissonii* and hybrids did occur (Fig. 1); only bees and bumblebees realized heterospecific transitions, but also very few in comparison to conspecific transitions. However, transitions between either species and hybrids occurred much more frequently (Fig. 2), with no bias towards any of the parental species (hybrids x *P. anisodora* vs. hybrids x *P. poissonii*: 66 vs. 63; χ^2^ = 0.09; *p* = 0.7). So we were able to reject the hypothesis that directions of hybridization and/or backcrossing was determined by the flight behavior of the pollinators.

### Hand pollination experiments

#### Between parental species

**Fig. 3:**
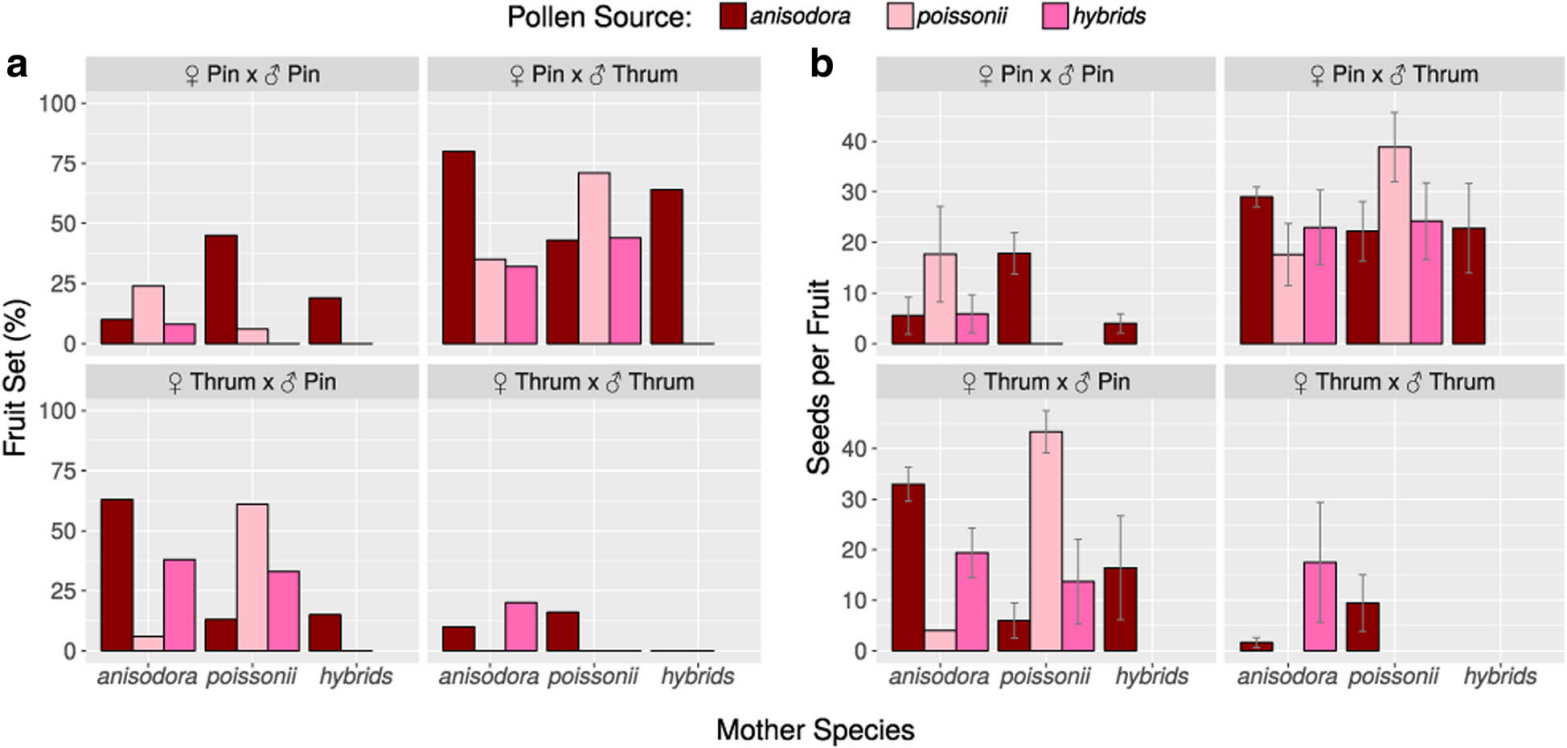
Fruit set (**a**) and seed numbers per fruits (**b**) after artificial cross-pollination in 2015 (*P. anisodora* x *P. poissonii*) and 2016 (*P. anisodora* x hybrids &*P. poissonii* x hybrids). Experiments are separated by cross type involving the different combinations of flower morphs (pin, thrum). For intra-morph crosses, incompatibility effects of the hetorostyly-supergene can be expected. Con-specific crosses produced more fruits/seeds in inter-morph crosses, with thrum/pin having a more pronounced pattern than pin/thrum, while hybrid pollen produced similar amounts of fruit/seeds for both species. For hybrid mothers fruits were only produced from pollination by *P. anisodora*

Results from fruit- and seed-set showed nearly exactly the same pattern: when a certain type of cross resulted more often in fruit set, that type of cross also resulted, on average, in higher seed numbers per fruit (Fig. 3a, b). Furthermore, both species produced comparable numbers of seed as there was no significant effect of the factor ‘Mother Species’ on fruit-set or seed-set (Table 1). As expected intra-morph (pin-pin, thrum-thrum) cross pollinations produced significantly lower number of fruits and seeds than inter-morph (pin-thrum, thrum-pin) cross pollinations (Fig. 3a, b; ‘Cross Type’ in Table 1). Thrum flowers seemed to be more selective, as they produced many fewer fruits/seeds from inter-specific pollen. Overall *P. anisodora* was the more successful pollen-parent, and this was most pronounced in intra-morph pollinations (Fig. 3a, b ‘Pollen Source’, ‘Pollen Source x Cross Type’, ‘Pollen Source x Mother Species’ and ‘Pollen Source x Flower Type’ in Table 1).

**Table 1:** ANOVAs for fruit set and seed numbers. Results of ANOVAs testing the contribution of several factors to seed production in pollination treatments carried out in 2015. Three stages were assessed: Fruit set (percentage of pollinations that resulted in fruit); Seed numbers per fruit; Seed number per flower. The following factors (with given levels) were included: Mother Species (anisodora, poissonii); Flower Type [of mother plant] (pin, thrum); Pollen Source (anisodora, poissonii); Cross Type (intra-morph, inter-morph). Significant results are highlighted in boldface. Nominator degrees of freedom (d. f.) = 1, denominator d. f. = 91

Factor	Fruit set		Seed number		Seeds per flower	
	*F-value*	*P*	*F-value*	*P*	*F-value*	*P*
*2015*						
Mother Species	0.009	0.924	0.446	0.506	0.733	0.394
Flower Type	**15.39**	**< 0.001**	**16.38**	**< 0.001**	**18.876**	**< 0.001**
Pollen Source	**7.279**	**0.008**	**6.073**	**0.016**	**11.126**	**0.001**
Cross Type	**51.043**	**< 0.001**	**62.398**	**< 0.001**	**81.533**	**< 0.001**
Mother Species × Flower Type	0.073	0.787	0.897	0.346	0.752	0.388
Mother Species × Pollen Source	**4.639**	**0.034**	**3.956**	**0.05**	3.286	0.073
Mother Species × Cross Type	0.091	0.764	3.522	0.064	1.164	0.284
Flower Type × Pollen Source	**8.029**	**0.006**	**16.526**	**< 0.001**	15.153	**< 0.001**
Flower Type × Cross Type	0.185	0.668	0.008	0.928	0.054	0.817
Pollen Source × Cross Type	**33.323**	**< 0.001**	**48.845**	**< 0.001**	57.518	**< 0.001**
Mother Species × Flower Type × Pollen Source	0.092	0.763	0.260	0.612	0.080	0.777
Mother Species × Flower Type × Cross Type	0.580	0.448	0.005	0.944	0.039	0.844
Mother Species × Pollen Source × Cross Type	0.657	0.42	0.797	0.183	1.261	0.264
Flower Type × Pollen Source × Cross Type	0.113	0.738	0.376	0.541	0.312	0.578
Mother Species × Flower Type × Pollen Source × Cross Type	0.012	0.915	0.032	0.859	0.154	0.695

The three-way ANOVA analysis for intra-specific crosses (8 treatments) suggested that inter-morph pollination can produce significantly higher numbers of fruits (F = 159.8, *p* < 0.001), seed numbers per fruits (F = 168.4, *p* < 0.001) and seed numbers per flower (F = 115.4, *p* < 0.001) than those produced from intra-morph pollination treatments (‘Cross type’ in Additional file 2: Table S2).

#### Between parental species and hybrids

In total, 7 of the 16 cross-pollination treatments between hybrids and parental species did not produce any fruits. Especially, *P. poissonii* pollen failed to produce any seed on hybrid mothers (Fig. 3a, b, ‘hybrids’), accounting for six of the failed pollination treatments (Additional file 1: Table S1). This resulted in significantly lower fruit-set and seed numbers following pollinations between hybrids and *P. poissonii* (16/143 = 11.2%) compared to hybrids and *P. anisodora* (37/168 = 22.0%), (fruit-set: χ^2^ = 5.67; *p* = 0.02); seed numbers (hybrids x *P. poissonii* 6.25 vs. hybrids x *P. anisodora* 10.75; Mann-Whitney U = 14, *p* = 0.048, Additional file 1: Table S1).

### Molecular results

#### Population genetic analysis

We genotyped 168 accessions for all 6 nuclear microsatellite loci screened across three allopatric populations and the hybrid zone: only two accessions failed to amplify at all loci (Ab17 for P39450–2 and AX9 for P28273). There was a single instance of evidence for linkage disequilibrium among the pairs of loci (P39450–2/P47381) in the hybrids in the Baishuitai population. There was one locus (P33802) displaying deviation from Hardy–Weinberg equilibrium (HWE) in the Xiaoyanjing population. As no consistent patterns of deviations from HWE or linkage equilibrium were detected across sites, downstream analyses were based on genotypic data at all microsatellite loci [14, 15].

Population genetic analysis showed that the putative hybrids had the highest numbers of effective alleles (2.136), and the highest observed (0.573), expected (0.511) and unbiased expected heterozygosity (0.518) (Additional file 4: Table S4). Pairwise F_ST_ values between parental species and putative hybrids among populations were highest between *P. poissonii* at Shangri-La and *P. anisodora* in Langdu (0.614) and lowest of *P. anisodora* between Baishuitai and Langdu populations (0.045). Within the hybrid zone in Baishuitai, the highest pairwise F_ST_ value was (0.472) between parental species whereas hybrids and *P. anisodora* has lowest F_ST_ value (0.119), and intermediate F_ST_ value (0.177) between hybrids and *P. poissonii* (Additional file 5: Table S5).

#### Parent and hybrid assignment

**Fig. 4:**
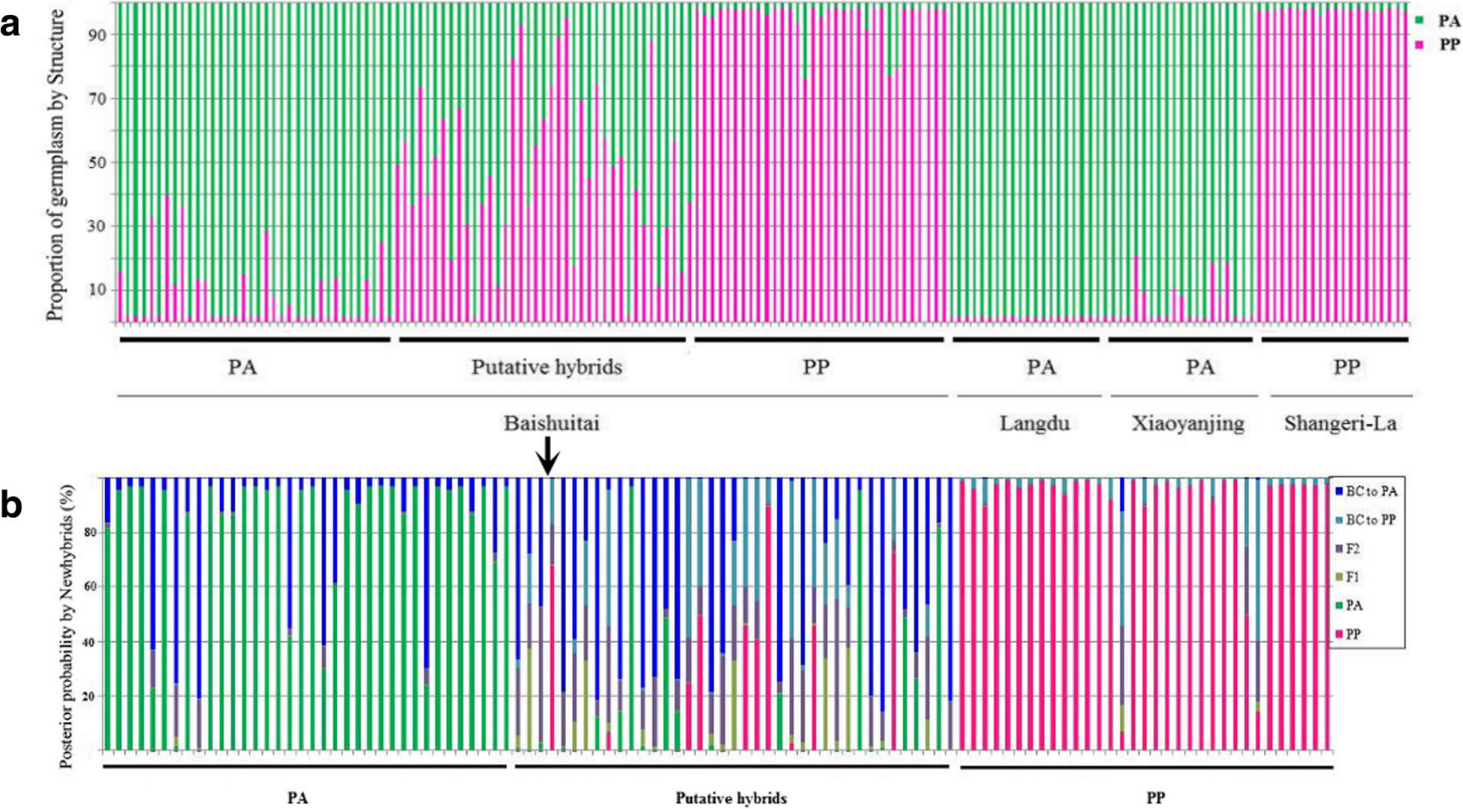
Genotype class assignment of all 168 samples based on the program Structure (**a**) and 108 samples in the hybrid zone by the program Newhybrids (**b**)

The value of ΔK was clearly highest for K = 2, and therefore we carried out all further analyses assuming two clusters (Additional file 7: Figure S2). Following ten independent Structure runs with K = 2, individuals of the Shangri-La population that had been identified morphologically as *P. poissonii* were assigned to one cluster with high probability, whereas those identified by us as *P. anisodora* had been assigned to the second cluster with similarly high probability (Fig. 4a). However, in the Xiaoyanjing population three accessions show certain amounts of mixed ancestry (*P. anisodora* cluster proportion < 90%), suggesting the occurrence of hybridization in the past. Within the Baishuitai hybrid zone, 13 accessions of *P. anisodora* and three accessions of *P. poissonii* show the same pattern, with the main cluster ancestry being < 90% indicative of introgression.

The proportion of backcrossed individuals of *P. anisodora*is higher than the proportion of backcrossed individuals of *P. poissonii*in the hybrid zone (13:3; χ^2^ = 5.67, df = 1, *p* = 0.018). Overall, two of the three investigated *P. anisodora* populations show signs of present or past introgression from *P. poissonii* (Fig. 4a).

The output from NewHybrids is in agreement with the clustering in Structure: the same 13 individuals of *P. anisodora* and three individuals of *P. poissonii* have posterior probabilities of less than 90% that they are not of hybrid origin (Fig. 4b). Hence, significantly more *P. anisodora* individuals show traces of introgression than *P. poissonii* (χ^2^ = 6.71, df = 1, *p* = 0.01). Of the 39 putative hybrids, two were identified as pure *P. anisodora* with posterior probabilities > 90%, whereas 30 as backcrosses to *P. anisodora* with posterior probabilities > 60%. The remaining hybrids, with any posterior probabilities< 60%, were all classified as later generation hybrids (Fig. 4b). Noticeably, no individuals in the hybrid zone were identified as F_1_ (in all cases the posterior probability for this class was under 30%).

Of the 150 individuals simulated from the Baishuitai data, 134 individuals were assigned to the correct class with > 90% probability, thus 89.4% assignment success was identified according to our SSRs. Of the F_1_s and simulated parental species, 100% were correctly assigned.

## Discussion

### Pre- and post- zygotic reproductive barriers to hybrid formation

In the sympatric hybrid zone in Baishuitai, the flowering periods of *P. poissonii* and *P. anisodora* overlapped completely. However, despite this no F1 s were detected with nearly 100% distinguishing power from hybrid simulations for this category.Part of this lack can likely be explained by the relatively high constancy of the pollinators (Fig. 2, conspecific vs. heterospecifc), combined with the observation that *P. poissonii* is largely pollinated by butterflies (Fig. 1), which generally do not visit *P. anisodora* at all. That this initial stage of hybridization can be expected to be rare is also visible from the overall ethological reproductive isolation indexes at the pollination stage in both *P. poissonii-* and *P. anisodora-*dominated parts of the population (Plot 1 and Plot 2), which were both above 0.94. Furthermore, certain intrinsic compatibilities between the species seem to exist, as conspecific pollinations produced significantly more fruit and seed than heterospecifc ones (Fig. 3; pin/thrum, thrum/pin), although considerable amounts of seed were produced, especially for pairings where intra-species-morph incompatibilities exist (Fig. 3, pin/pin).

Different isolating barriers act in hierarchical order, and thus early barriers can contribute more to total isolation than late barriers [16, 17], suggesting a key role for pollinator-mediated reproductive isolation between parental species, as evidenced by the high ethological reproductive isolation. We assume that the different flower colors (deep-red vs. magenta) are differently attractive for the two major pollinator groups (bees/bumblebees vs. butterflies), but additionally the flower orientation in *P. anisodora* (drooping) makes it very difficult for butterflies to access them. This results in bees and bumblebees preferring *P. anisodora*, but occasionally visiting *P. poissonii*, while butterflies are restricted to *P. poissonii*. However, as no mechanical barrier exists, transition rates of bees/bumblebees could be locally altered by context-dependent selection, as exemplified by hybridization patterns between *Rhinanthus minor* and *R. angustifolius* [18]. Although the initial formation of F_1_s is rare, as soon as some hybrids exist, further hybridization seems to be facilitated by partial breakdown of pollinator discrimination, as pollinators showed many more transitions between hybrids and parental species than between parental species (Fig. 2, heterospecifc vs. hybrid).

### Highly asymmetric introgression

The genetic data provided evidence for significant introgression into *P. anisodora*, while only few *P. poissonii* individuals showed such signs (Fig. 4a, b). Asymmetry in backcrossing is not uncommon in nature, assuming asymmetric strength of reproductive barriers in both pre- and post-zygotic stages - it can even be expected [4, 19–22]. With regards to pre-zygotic isolation, no significant difference between transition rates from hybrids to *P. anisodora* (66) and hybrids to *P. poissonii* (63) was apparent in the observed pollinators, and hence is unlikely to have an effect on the direction of backcrossing. On the other hand, intrinsic compatibilities seemed to favor backcrossing to *P. anisodora,* ashybrid mothers were only successfully fertilized by *P. anisodora* pollen. It should be noted that as no F_1_s were detected in the hybrid zone, most hybrids that were included in the pollinator observations and hand pollination experiments were probable backcrosses to *P. anisodora*and potentially other types of later generation hybrids, but likely with reduced *P. poissonii* heritage. This might have led to comparably lower RI between hybrids in our study and *P. anisodora* plants, than would have been detected if F_1_s had been involved, but does not entirely explain why hybrids did not produce fruit from *P. poissonii* pollen, as *P. anisodora* plants could be fertilized.

Additionally, one factor contributing to the asymmetry could be the differences in abundance of the two species: many more flowering plants of *P. poissonii* than *P. anisodora* were present in the population in Baishuitai, reaching a ratio of nearly 4:1 in the sympatric area (in 2017). Assuming that some F_1_s have been formed, and that pollinators distinguish well between species but worse between hybrids and either of the species, abundance differences in the parental species would lead to a skew just by sampling bias. E.g. for a ratio of 4:1 (*P. poissonii* to *P. anisodora*), and including 4% hybrids or: 76.8% *P. poissonii*, 19.2% *P. anisodora*, 4% hybrids, assuming pollen will originate from the species and hybrids with equal likelihood, hybrid pollen (as compared to conspecific pollen) being deposited on *P. poissonii* and *P. anisodora* flowers would be 4 / (4 + 76.8) = 4.95%, and 4 / (4 + 19.2) = 17.2%, respectively. Furthermore, for hybrids, only pollen from *P. anisodora* would lead to fertilization, reinforcing the bias.

### Conservation management of *P. anisodora* to prevent genetic swamping

As *P. anisodora* has only three currently known wild populations, and it is known to hybridize with a widely distributed species, conservation may be necessary to prevent genetic swamping. According to our results, the population of *P. anisodora* in Baishuitai was severely affected by introgression of *P. poissonii* markers, indicating that a threat of genetic swamping might exist. In a second population in Xiaoyanjing, in which no other *Primula* species were found, only four individuals out of 20 showed possible introgression of *P. poissonii*. The last sampled population of *P. anisodora,* in Langdu, had no evidence of any past introgression, and we suggest that urgent conservation action should be taken to protect this population.

Guidelines have been recently proposed outlining action when hybridization involves endangered species [9, 10]. The first thing necessary to clarify is whether the hybridization occurred naturally or was induced anthropogenically [10]. We consider that the hybridization between *P. poissonii* and *P. anisodora* was probably induced by human activity, because a new road going through the hybrid zone was constructed in the 2000s, and close by, hotels have been built to attract tourists. With greater human population pressure, grazing by livestock becomes a more serious threat. For instance, during the periods of the field experiment, it was common to see cows eating the leaves and flowers of *P. anisodora*. Furthermore, all putative hybrids were growing together with parental species and we did not observe any hybrids occupying new habitats, indicating potential competition for the remaining habitat. In contrast, the “pure” *P. anisodora* population in Langdu showed no evidence of human activity and is situated in a remote area far from any villages. The remaining population of *P. anisodora* in Xiaoyanjing, while far from any villages, is very close to an asphalt road, and hence might be under threat.

Furthermore, the present study suggests that hybrids interfere with pollinator-mediated RI, and can lead to an acceleration of the invasion of the genepool of *P. anisodora*, which can be especially dangerous in situations where *P. poissonii* is more frequent. While the first step towards hybridization (F_1_s) seems to be a difficult one, later backcrossing seems to occur with more ease.

Therefore, based on our main findings, we recommend the following conservation action plan for *P. anisodora*. (i) Given the road going through the hybrid zone in Baishuitai, two protective plots should be set separately. The first plot will be above the road and very close to the local village. This plot should be protected because the largest number of *P. anisodora* individuals can be found here. The second plot, below the road, would include some *P. anisodora*, some hybrids and many individuals of *P. poissonii*. It would be preferable to conduct these actions with the help and support of the local people, rather than enforcing protection by strict regulation. (ii) Over the course of several years populations should be screened for hybrids, at best during the flowering season, and all plants resembling morphological hybrids in both plots should be removed. Thus a sympatric area without flowering hybrids would be recovered. We would expect that much stronger reproductive isolation will be maintained between *P. poissonii* and *P. anisodora* in such a setting. (iii) A detailed field investigation should be carried out in Xiaoyanjing to verify whether or not any other *P. poissonii* populations occur on the same mountain. Both species grow along streams, and seed can be easily dispersed by water. Hence if a *P. poissonii* population exists even some distance away from the *P. anisodora* population, a sympatric area can form if a stream is shared. If no *P. poissonii* is found, no conservation action needs to be undertaken here, as we would expect that after successive generations of backcrosses between those *P. anisodora* plants with *P. poissonii* genetic material and *P. anisodora*, all individuals here will eventually become almost pure *P. anisodora*. (iv) Given that the population of *P. anisodora* in Langdu is most strongly differentiated from the other two populations (F_ST_ for Langdu & Xiaoyanjing = 0.433; F_ST_ for Langdu & Baishuitai = 0.347; F_ST_ for Baishuitai & Xiaoyanjing = 0.045), this population could be used to reinforce genetic diversity in the other populations. To this end, seed should be collected, and plants propagated before introduction to the other two populations.

## Conclusions

We investigated hybridization and reproductive barriers between a wide spread (*P. poissonii*) and a rare promise species (*P. anisodora*). Microsatellites analysis for the hybridizing species clarified the occurrence of backcrosses to *P. anisodora* in two of the three populations of *P. anisodora,* and more individuals of *P. anisodora* showed introgression from *P. poissonii* than vice versa in the sympatric area. Pollinator observations revealed that pollinator-mediated reproductive isolation was found to be still strong between parental species but not significant between hybrids and parental species. Hand pollination treatments showed intrinsic incompatibilities between parental species and moreover, hybrids had higher fruit- and seed-set when pollinated with *P. anisodora* pollen as opposed to *P. poissonii* pollen, indicating that asymmetric hybrid incompatibility might also play roles for the genetic structure of the hybrid zone. Overall evidence of potential genetic swamping of the *P. anisodora* populations was detected in the present study, and detailed conservation managements to reduce the risk by genetic swamping to *P. anisodora* were advised.

## Methods

### Plant species, study site and sampling

The two study species *P. poissonii* and *P. anisodora* are perennial herbs, growing on alpine meadows at altitudes between 2500 and 3700 m [23]. While *P. poissonii* is relatively widespread, occurring in the eastern Himalayas (Tibet, Yunnan and Sichuan), *P. anisodora* is only known from three populations in Yunnan province (China): two populations (Langduand Baishuitai) in Shangri-La county and one population (Xiaoyanjing) in Nujiang county (Additional file 6: Figure S1). We only know of one area where both species grow in sympatry and seem to form hybrids (Baishuitai). This hybrid zone spans an area of about 15 km^2^ in Baishuitai, Shangri-La, NW Yunnan (27.6°N, 100.04°W, 3100 m). The population is situated in wet alpine meadow, with a stream running through it. In 2017, individual counts for *P. anisodora* and *P. poissonii* were 517 and 2349 flowering plants, respectively.

**Fig. 5:**
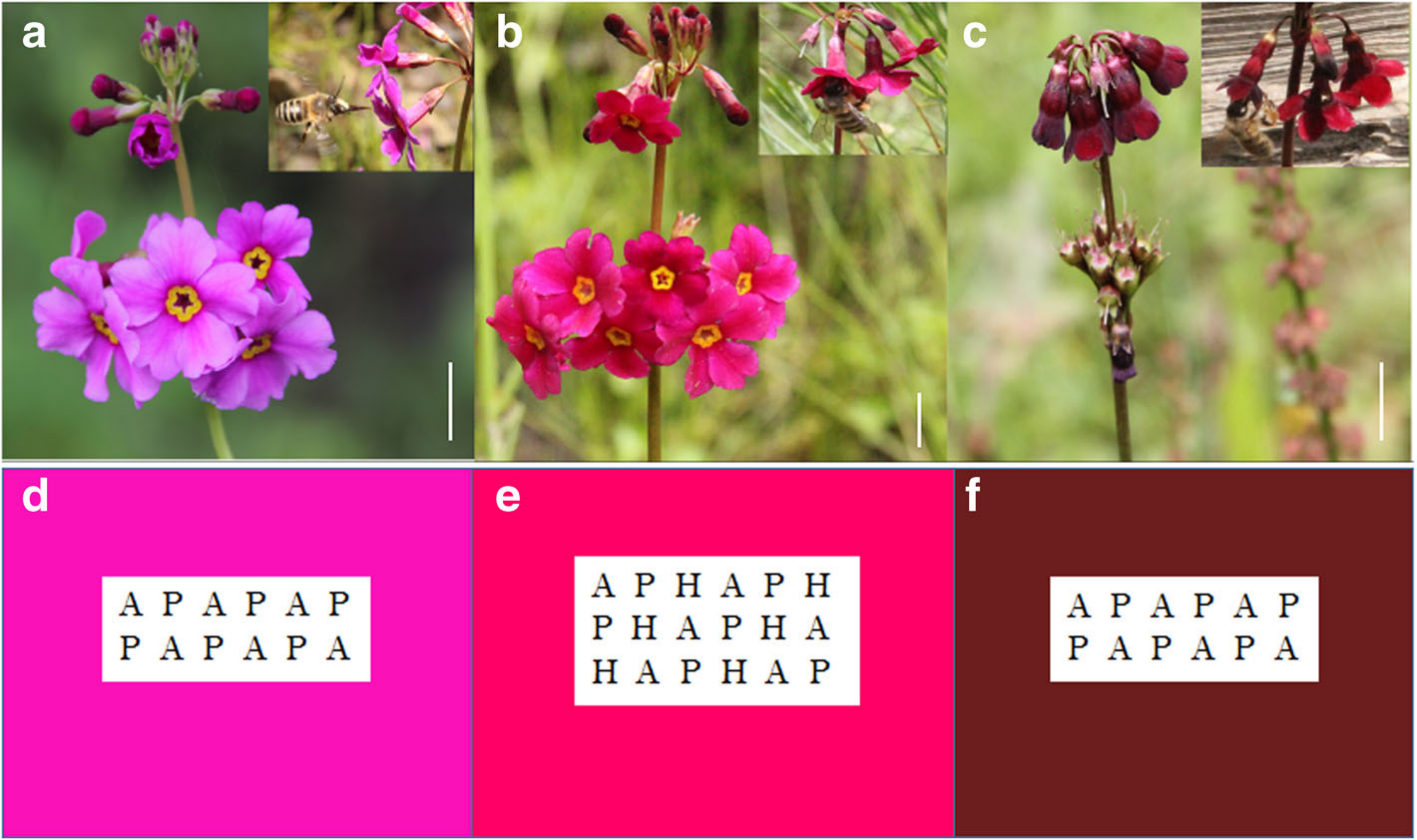
Flower morphology and pollinator observation arrays. **a**, **b**, **c** Flower characteristics of *P. poissonii*, hybrids and *P. anisodora* with their main shared pollinators in upper-left. **d**, **e**, **f** Pollinator transition observations of arranged species in *P. poissonii*, hybrids and *P. anisodora* dominant plots. Bars, 10 mm

Parental species and hybrids can be easily distinguished by flower color and floral orientation: *P. anisodora* has dark-red, drooping flowers; *P. poissonii* has magenta-colored flowers that are oriented perpendicular to the stem; putative hybrids have intermediate color and floral orientation (Fig. 5a, b, c).

During the flowering period in 2014, a total of 168 individuals were sampled: 108 from the hybrid zone (*P. anisodora*: 36 individuals; *P. poissonii*: 33 individuals; putative hybrids: 39 individuals); 20 each from the two allopatric *P. anisodora* populations - Xiaoyanjing (26.58°N, 99.44°W), Langdu (27.95°N, 99.7°W); and 20 *P. poissonii* from an allopatric reference population near Shangri-La county (28.2°N, 100.04°W). Leaves were collected and preserved in silica gel in individually numbered containers. We stated here that all samples collected and used in this study as well as pollination experiments performed below in the field do not need any permission. We declare plant materials used in the current research complied with government regulations.

### Reproductive barriers in the hybrid zone

The flowering times of the two species overlap completely, starting early in July, and ending in mid August. Therefore we predicted that pollinator-mediated isolation and intrinsic incompatibilities could play a role for isolating parental species and hybrids, and might also determine hybridization patterns in the sympatric area.

#### Pollinator-mediated reproductive isolation

Pollinator observations were carried out in two types of setting: (1) natural setting - from 6 July to 10 July in 2015 pollinators were observed in natural either *P. poissonii-* or *P. anisodora-*dominated parts of the population, to assess their pollinator assemblages; and (2) artificially controlled plot setting - three plots were established within the hybrid zone to evaluate reproductive barriers between parental species. The first of these plots (Plot 1) was set up in a *P. poissonii-*dominated part of the population and comprised both parental species (10 plants each) in an alternating setup (Fig. 5d); Plot 2 had an identical layout to plot 1, but was placed in a *P. anisodora-*dominated part of the population (Fig. 5f); Plot 3 was placed inside the hybrid zone, and comprised both parents and hybrids (Fig. 5e). Individual plants in the grid setups were placed approximately 50 cm apart, and species / hybrid identity was assigned according to morphological characters. From 6 July to 12 July in 2015, both Plot 1 and Plot 2 were observed for 7 days; each observation day lasting from 09:00 to 17:00. However, if weather conditions were unfavorable (rain) for pollinators, field observations were stopped during those hours. Plot 3 was observed on five sunny days from 7 to 11 July, 2015. We recorded a flower approach by a pollinator as a visit only if the pollinator had foraged or interacted with the flower by touching the reproductive structures of the plant. All types of flower visitors were photo recorded, identified, and preserved in the insect collections of Kunming Institute of Botany, CAS.

Following Natalis and Wesselingh [18], we calculated overall ethological reproductive isolation (RI) in *P. poissonii-* as well as *P. anisodora-*dominated parts of the population as: 1 - (No. cross-species foraging bouts / total number of foraging bouts) × (heterospecific transitions / all transitions). Herein one bout means the visitation of an insect to the plot, from approaching the plot up to its departure, irrespective of how many plants within the plot were visited, whereas transition refers to the type (conspecific vs heterospecifc) and number of visitations within one bout.

#### Hand pollination experiments

Pollination experiments were carried out in 2015 and 2016. The main focus between the years was different: in 2015 cross-compatibility between the species was tested, and in 2016 fertility between hybrids and parental species was investigated. Although it would have been desirable to carry out both of the experiments in either year, this was not possible, as the number of flowering plants of *P. anisodora* would not allow appropriate sample sizes for both experiments in the same year.

#### Cross-compatibility of the parental species

In July 2015, cross-pollination experiments were carried out for 16 possible combinations between the flower-morph types and species. Because both species are heterostylous, four intra specific pollination treatments are possible (♀-♂): P-P; P-T; T-P; T-T. These same four treatments also apply to inter-specific cross-pollination, and hence 16 treatments are necessary to cover all possible combinations.

For each treatment a minimum of 30 plants were selected as mothers. Up to three flowers per plant were selected while in bud, and the rest removed mechanically; afterwards the plant was labeled and bagged with a nylon net. When the flowers had just opened, flowers of the respective pollen parent were collected, and recipient flowers were pollinated manually by brushing the anthers against the stigma. After application of pollen the nylon net-bag was replaced and left for a further 3 days. In September 2015, fruits were harvested, and seed numbers per fruit were counted. Several plants were damaged by grazing before they could be harvested, resulting in 15 to 35 flowers per treatment that could be assessed and which are given as sample size ( Additional file 1: Table S1).

#### Fertility between hybrids and parental species

In July 2016, we focused on barriers to backcrossing between hybrids and parental species. To this end we performed cross-pollinations between hybrids and the parental species. As before, the different plant morph types were treated separately, resulting in 16 treatment combinations (Additional file 1: Table S1). The experiment was carried out as in 2015, but for some treatments grazing damage was higher, resulting in assessable sample sizes between 12 and 33 pollinated flowers. Fruits were again harvested in September, 2016.

### Data analysis

To detect differences of visitation percentages between parental species, a χ^2^ test was employed. For assessing the effect of factors below on fruit- and seed-set for the pollination treatment data we employed ANOVAs as implemented in SPSS 15.0 for Windows (Chicago, IL, USA).

For the 2015 data we included four factors: Mother Species (anisodora, poissonii); Flower Type of mother plant (pin, thrum); Pollen Source (anisodora, poissonii); and Cross Type (intra-morph, inter-morph). Additionally a three-way ANOVA analysis for intra-specific pollination treatments was done including the factors Mother Species, Flower Type and Pollen Source. In order to improve ANOVA assumptions of normality and homogeneity, fruit sets were arcsine square-root transformed whereas seed numbers per fruit and seed numbers per flower were both log (x + 1) transformed.

For the 2016 data 7 treatments did not produce any fruits. We first compared differences in fruit sets and seed numbers between cross-pollinations of *P. poissonii* x hybrids and *P. anisodora* x hybrids using a χ^2^ test (fruit sets for pollination treatments of *P. poissonii* x hybrids and *P. anisodora* x hybrids were calculated as the total number of fruits divided by the total number of flowers within each treatment) and a Mann-Whitney nonparametric test (mean seed numbers per treatment as replicates, thus both *P. poissonii* x hybrids and *P. anisodora* x hybrids cross-pollinations comprising 8 replicates).

### Molecular analyses

#### DNA extraction and microsatellite genotyping

We extracted genomic DNA from the dried leaf tissue using a modified cetyl trimethyl ammonium (CTAB) protocol. Quantification of DNA was carried out with a SmartSpecTM Plus Spectrophotometer (Bio-Rad). To select candidate diagnostic microsatellites to identify parental species and hybrids, 219 SSR primers were designed based on sequences previously obtained on a MiSeq Benchtop Sequencer (Illumina, Inc., San Diego, CA, USA) for the close relative *P. chungensis* [24]. Detailed protocols for PCR amplification and condition regarding these primers followed Zhou et al. [24]. PCR products were directly analyzed on a 3730xl Sequence Analyzer (Applied Biosystems, Foster City, CA, USA), using a LIZ GeneScan-500 size standard. Resulting chromatograms were visualized and converted to diploid genotypes using automated allele-calling implemented in GENEMARKER v.4.0 (SoftGenetics LLC, State College, PA, USA). All automated genotyping was re-checked manually. All genotypes for each locus and individual were entered into an Excel file following the format of GenALEx 6.5 [25].

#### Population genetic analysis

All basic summary statistics for the microsatellite data, and an AMOVA were calculated using GenALEx 6.5. Deviation from Hardy–Weinberg equilibrium (HWE) for each locus, and linkage disequilibrium (LD) for all loci pairs, were assessed with the web version of GENEPOP, v4.0.10 [26]. For the HW test the heterozygote deficiency option was used. For the LD test the following Markov Chain parameters were set: 5000 dememorization runs; 1000 batches each with 5000 iterations.

#### Parent and hybrid assignment

To estimate parental ancestry proportions for each hybrid, microsatellite data was analyzed using the program STRUCTURE version 2.3.1 [27]. We adopted the admixture model with correlated allele frequencies [19]. No prior knowledge of the species was included in the analyzed data set. To determine the optimal number of groups (K), we ran STRUCTURE with K varying from 1 to 10, with five runs for each K value, and following Evanno et al. (2005) used ΔK to choose the most likely number of clusters. MCMC runs were then performed using a burn-in of 10,000 followed by 10,000 iterations.

Hybrid class assessment based on the microsatellite data was carried out with the program NewHybrids [28], using the following settings: burn-in of 10,000 followed by 100,000 MCMC iterations with the Uniform priorsmode. For the classification of individuals into each of the three groups, a relatively strict posterior probability of 0.9 was used, and only individuals reaching such a probability were taken as being classified; others were considered not properly classified by the program.

#### Hybrid swarm simulation

As the analyses were based on six loci only, we assessed their discriminatory power in NewHybrids by simulating several classes of hybrids based on parental individuals that had membership to either cluster in STRUCTURE close to 1 (‘pure’ parents). We simulated 5 hybrid categories (two parental species, F_1_and the first backcross to each parent) from 23 each of pure *P. anisodora* and *P. poissonii* (both > 90% probability in both structure and NewHybrids) using HYBRIDLAB version 1.0 [29]. For each category, 30 individuals were generated, hence a total of 150 individuals (30 of each parent, 30 F_1_s and 30 BC to each parent) were simulated for the hybrid swarm in Baishuitai, and these were subsequently analyzed in NewHybrids.

## Additional files

Additional file 1: **Table S1.** Fruit set and seed numbers per fruits from 16 pollination treatmentswithin and between parental species in 2015 and between hybrids and parental species in 2016, with *n* referring to sample size and *na* to not available when no seeds were found in fruit. (DOCX 24 kb)

Additional file 2: **Table S2.** The three-way ANOVA analysis for intra-specific crosses (8 treatments) in 2015.Significant results are highlighted in boldface. (DOCX 14 kb)

Additional file 3: **Table S3.** Numbers of transitions of different pollinators in plot 3. For these 9 types of transitions, the first plant is the currently visited species and the second plant is the species that the pollinator’s next approaching to. PA, *P. anisodora*; PP, *P. poissonii* and PH, putative hybrids. (DOCX 14 kb)

Additional file 4: **Table S4.** Sample number (N), mean allele number per locus (Na), number of effective alleles (Ne), information index (I), observed heterozygosity (Ho), expected(He) and unbiased expected (uHe) heterozygosity of microsatellite loci in each population of *P. anisodora*, *P. poissonii* and hybrids. (DOCX 14 kb)

Additional file 5: **Table S5.** Pairwise F_st_ values between parental species and putative hybrids among populations. (DOCX 14 kb)

Additional file 6: **Figure S1.** Basic information on all known populations of *P. poissonii* and *P. anisodora* as well as our sampling and experimental locations; with 1 = Langdu population, 2 = Shangeri-La, 3 = Baishuitai, 4 = Xiaoyanjing. The distribution maps are plotted based on the species distribution data at the county level supplied by the Chinese Virtual Herbarium (http://www.cvh.ac.cn/). (DOCX 189 kb)

Additional file 7: **Figure S2.** ΔK values for the Structure analysis. (DOCX 34 kb)

## Data Availability

The data sets supporting the results of the present study are included within this article (and its additional files).

## Abbreviations

ANOVA: Analysis of Variance
RI: Reproductive isolation
SSR: Simple sequence repeat
