# Supplementary material for: Conservation implications of asymmetric introgression and reproductive barriers in a rare primrose species
Source: BMC Plant Biol. 2019 Jun 28;19:286. doi: 10.1186/s12870-019-1881-0 (PMC6599365; doi:10.1186/s12870-019-1881-0)
Supplement: Supplementary file 1 — Table S1. Fruit set and seed numbers per fruits from 16 pollination treatmentswithin and between parental species in 2015 and between hybrids and parental species in 2016, with n referring to sample size and na to not available when no seeds were found in fruit. (DOCX 24 kb) [file 12870_2019_1881_MOESM1_ESM.docx]

**Additional file 1: Table S1** Fruit set and seed numbers per fruits from 16 pollination treatmentswithin and between parental species in 2015 and between hybrids and parental species in 2016, with *n* referring to sample size and *na* to not available when no seeds were found in fruit.

|  | Treatment | Fruit set | | | | | |  | Seeds per fruit | | | | | |
| --- | --- | --- | --- | --- | --- | --- | --- | --- | --- | --- | --- | --- | --- | --- |
| Maternal species | MxF | Intra-specific | n | Inter-specific | n | hybrids | n |  | Intra-specific | n | Inter-specific | n | hybrids | n |
| *P. poissonii* | PxP | 0.06 | 35 | 0.45 | 20 | 0 | 16 |  | 0 | 2 | 17.8±4.08 | 9 | na | 0 |
|  | TxT | 0 | 30 | 0.16 | 31 | 0 | 19 |  | na | 0 | 9.44±5.59 | 5 | na | 0 |
|  | PxT | 0.71 | 24 | 0.43 | 30 | 0.44 | 27 |  | 38.86±6.86 | 17 | 22.2±5.89 | 13 | 24.2 ± 7.6 | 12 |
|  | TxP | 0.61 | 23 | 0.13 | 30 | 0.33 | 12 |  | 43.33±4.20 | 14 | 6±3.47 | 4 | 13.7 ± 8.4 | 4 |
| *P. anisodora* | PxP | 0.1 | 21 | 0.24 | 21 | 0.08 | 26 |  | 5.55±3.67 | 2 | 17.67±9.41 | 5 | 5.9± 3.8 | 2 |
|  | TxT | 0.1 | 20 | 0 | 26 | 0.2 | 20 |  | 1.6±0.98 | 2 | na | 0 | 17.5 ± 11.9 | 4 |
|  | PxT | 0.8 | 15 | 0.35 | 20 | 0.32 | 31 |  | 29±1.98 | 12 | 17.6±6.12 | 7 | 22.95 ± 7.4 | 10 |
|  | TxP | 0.63 | 16 | 0.06 | 16 | 0.38 | 13 |  | 33±3.32 | 10 | 4 | 1 | 19.4± 4.9 | 5 |
|  |  |  |  |  | | | |  |  | | | |  |  |
|  |  | Pollen source |  | *P. poissonii* |  | *P. anisodora* |  |  |  |  | *P. poissonii* |  | *P. anisodora* |  |
| Hybrids | PxP |  |  | 0 | 15 | 0.19 | 21 |  |  |  | na | 0 | 4 ± 1.9 | 4 |
|  | TxT |  |  | 0 | 18 | 0 | 23 |  |  |  | na | 0 | na | 0 |
|  | PxT |  |  | 0 | 19 | 0.64 | 14 |  |  |  | na | 0 | 22.8 ± 8.9 | 9 |
|  | TxP |  |  | 0 | 17 | 0.15 | 20 |  |  |  | na | 0 | 16.4 ± 10.3 | 3 |
