# Supplementary material for: Conservation implications of asymmetric introgression and reproductive barriers in a rare primrose species
Source: BMC Plant Biol. 2019 Jun 28;19:286. doi: 10.1186/s12870-019-1881-0 (PMC6599365; doi:10.1186/s12870-019-1881-0)
Supplement: Supplementary file 2 — Table S2. The three-way ANOVA analysis for intra-specific crosses (8 treatments) in 2015.Significant results are highlighted in boldface. (DOCX 14 kb) [file 12870_2019_1881_MOESM2_ESM.docx]

**Additional file 2: Table S2** The three-way ANOVA analysis for intra-specific crosses (8 treatments) in 2015.Significant results are highlighted in boldface.

| Source （2015） | Fruit set | | Seed number | | Seed number per flower | |
| --- | --- | --- | --- | --- | --- | --- |
|  | *F-value* | *P* | *F-value* | *P* | *F-value* | *P* |
| Mother species | 2.582 | 0.115 | 1.539 | 0.221 | 0.472 | 0.495 |
| Flower type | 1.669 | 0.203 | 0.186 | 0.668 | 0.015 | 0.902 |
| Cross type | **159.751** | **<0.001** | **168.351** | **<0.001** | **115.36** | **<0.001** |
| Mother species × Flower type | 0.028 | 0.869 | 0.17 | 0.682 | 0.035 | 0.852 |
| Mother species × Cross type | 0.065 | 0.799 | 6.679 | **0.013** | 1.443 | 0.236 |
| Flower type × Cross type | 0.816 | 0.371 | 1.368 | 0.248 | 0.025 | 0.875 |
| Mother species × Flower type × Cross type | 0.453 | 0.504 | 0.106 | 0.746 | 0.009 | 0.925 |
