# Supplementary material for: Conservation implications of asymmetric introgression and reproductive barriers in a rare primrose species
Source: BMC Plant Biol. 2019 Jun 28;19:286. doi: 10.1186/s12870-019-1881-0 (PMC6599365; doi:10.1186/s12870-019-1881-0)
Supplement: Supplementary file 3 — Table S3. Numbers of transitions of different pollinators in plot 3. For these 9 types of transitions, the first plant is the currently visited species and the second plant is the species that the pollinator’s next approaching to. PA, P. anisodora; PP, P. poissonii and PH, putative hybrids. (DOCX 14 kb) [file 12870_2019_1881_MOESM3_ESM.docx]

**Additional file 3: Table S3** Numbers of transitions of different pollinators in plot 3. For these 9 types of transitions, the first plant is the currently visited species and the second plant is the species that the pollinator’s next approaching to. PA, *P. anisodora*; PP, *P. poissonii* and PH, putative hybrids.

| Pollinators | PA-PA | PA-PP | PA-PH | PP-PP | PP-PA | PP-PH | PH-PH | PH-PA | PH-PP | Total |
| --- | --- | --- | --- | --- | --- | --- | --- | --- | --- | --- |
| *Anthophora sp.* | 120 | 8 | 21 | 33 | 12 | 20 | 28 | 23 | 14 | 279 |
| *Aporia bieti* | 5 | 0 | 0 | 89 | 0 | 13 | 3 | 0 | 11 | 121 |
| Bumblebees | 93 | 4 | 14 | 14 | 4 | 3 | 19 | 8 | 2 | 161 |
| Total | 218 | 12 | 35 | 136 | 16 | 36 | 50 | 31 | 27 | 561 |
