# Supplementary material for: Conservation implications of asymmetric introgression and reproductive barriers in a rare primrose species
Source: BMC Plant Biol. 2019 Jun 28;19:286. doi: 10.1186/s12870-019-1881-0 (PMC6599365; doi:10.1186/s12870-019-1881-0)
Supplement: Supplementary file 4 — Table S4. Sample number (N), mean allele number per locus (Na), number of effective alleles (Ne), information index (I), observed heterozygosity (Ho), expected(He) and unbiased expected (uHe) heterozygosity of microsatellite loci in each population of P. anisodora, P. poissonii and hybrids. (DOCX 14 kb) [file 12870_2019_1881_MOESM4_ESM.docx]

**Additional file 4: Table S4** Sample number (N), mean allele number per locus (Na), number of effective alleles (Ne), information index (I), observed heterozygosity (Ho), expected(He) and unbiased expected (uHe) heterozygosity of microsatellite loci in each population of *P. anisodora*, *P. poissonii* and hybrids.

| Species | Pop | N | Na | Ne | I | Ho | He | uHe |
| --- | --- | --- | --- | --- | --- | --- | --- | --- |
| *P. anisodora* | Baishuitai | 36 | 2.833 | 1.456 | 0.48 | 0.301 | 0.273 | 0.276 |
| Hybrids | Baishuitai | 39 | 2.667 | 2.136 | 0.803 | 0.573 | 0.511 | 0.518 |
| *P. poissonii* | Baishuitai | 33 | 2.333 | 1.59 | 0.485 | 0.333 | 0.291 | 0.295 |
| *P. anisodora* | Langdu | 20 | 1.333 | 1.329 | 0.23 | 0.308 | 0.166 | 0.17 |
| *P. anisodora* | Xiaoyanjing | 20 | 2.667 | 1.703 | 0.567 | 0.311 | 0.327 | 0.336 |
| *P. poissonii* | Shangeri-la | 20 | 2.167 | 1.455 | 0.418 | 0.275 | 0.256 | 0.262 |
