# Supplementary material for: Conservation implications of asymmetric introgression and reproductive barriers in a rare primrose species
Source: BMC Plant Biol. 2019 Jun 28;19:286. doi: 10.1186/s12870-019-1881-0 (PMC6599365; doi:10.1186/s12870-019-1881-0)
Supplement: Supplementary file 5 — Table S5. Pairwise Fst values between parental species and putative hybrids among populations. (DOCX 14 kb) [file 12870_2019_1881_MOESM5_ESM.docx]

**Additional file 5: Table S5**Pairwise F_st_ values between parental species and putative hybrids among populations

| *P. anisodora*  Baishuitai | Hybrids Baishuitai | *P.poissonii*  Baishuitai | *P.anisodora*  Langdu | *P.anisodora*  Xiaoyanjing | *P.poissonii*  *Shangerri-la* |  |
| --- | --- | --- | --- | --- | --- | --- |
| 0 |  |  |  |  |  | *P. anisodora* Baishuitai |
|  |  |  |  |  |  |  |
| 0.119 | 0 |  |  |  |  | *Hybrids Baishuitai* |
| 0.472 | 0.177 | 0 |  |  |  | *P.poissonii* Baishuitai |
|  |  |  |  |  |  |  |
| 0.045 | 0.207 | 0.582 | 0 |  |  | *P.anisodora* Langdu |
|  |  |  |  |  |  |  |
| 0.347 | 0.256 | 0.384 | 0.433 | 0 |  | *P.anisodora* Xiaoyanjing |
|  |  |  |  |  |  |  |
| 0.508 | 0.224 | 0.05 | 0.614 | 0.424 | 0 | *P.poissonii* Shangerri-la |
|  |  |  |  |  |  |  |
