# Supplementary material for: Conservation implications of asymmetric introgression and reproductive barriers in a rare primrose species
Source: BMC Plant Biol. 2019 Jun 28;19:286. doi: 10.1186/s12870-019-1881-0 (PMC6599365; doi:10.1186/s12870-019-1881-0)

**Additional file 6: Figure s1** Basic information on all known populations of *P. poissonii* and *P. anisodora* as well as our sampling and experimental locations; with 1 = Langdu population, 2 = Shangeri-La, 3=Baishuitai, 4= Xiaoyanjing. The distribution maps are plotted based on the species distribution data at the county level supplied by the Chinese Virtual Herbarium

(<http://www.cvh.ac.cn/>).


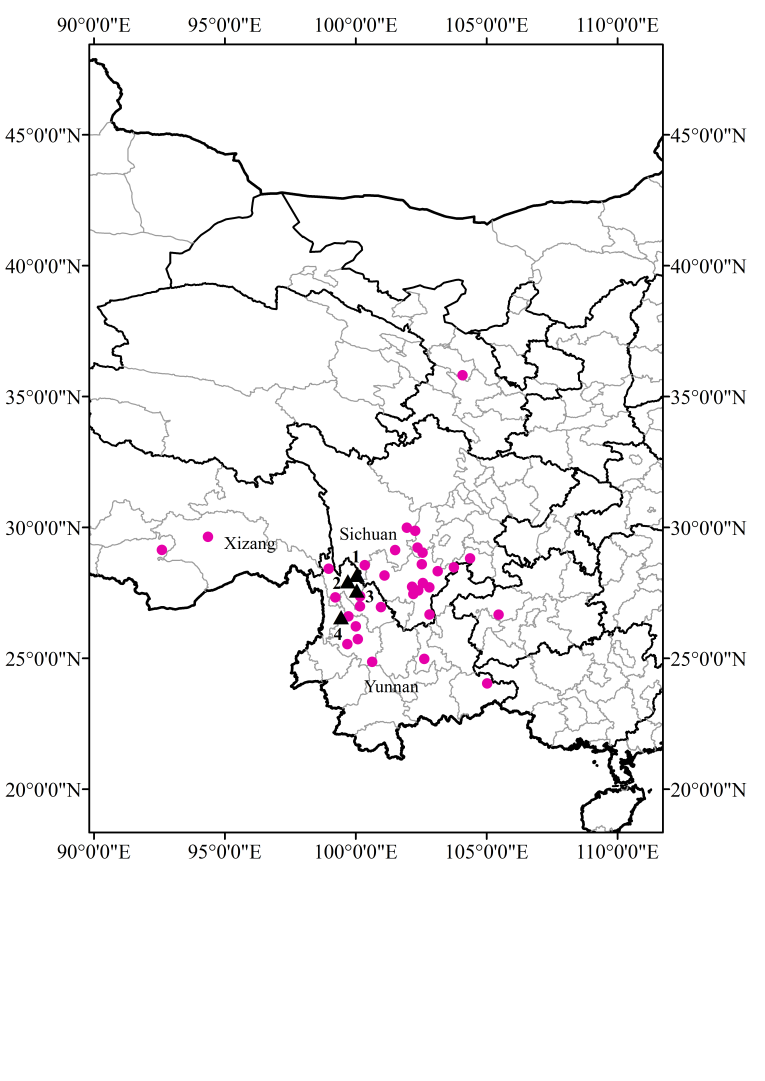

Supplement: Supplementary file 6 — Figure S1. Basic information on all known populations of P. poissonii and P. anisodora as well as our sampling and experimental locations; with 1 = Langdu population, 2 = Shangeri-La, 3 = Baishuitai, 4 = Xiaoyanjing. The distribution maps are plotted based on the species distribution data at the county level supplied by the Chinese Virtual Herbarium (http://www.cvh.ac.cn/). (DOCX 189 kb) [file 12870_2019_1881_MOESM6_ESM.docx]
