# Supplementary figures and images for: Conservation implications of asymmetric introgression and reproductive barriers in a rare primrose species
Source: BMC Plant Biol. 2019 Jun 28;19:286. doi: 10.1186/s12870-019-1881-0 (PMC6599365; doi:10.1186/s12870-019-1881-0)

**Additional file 7: Figure s2** ΔK values for the Structure analysis.


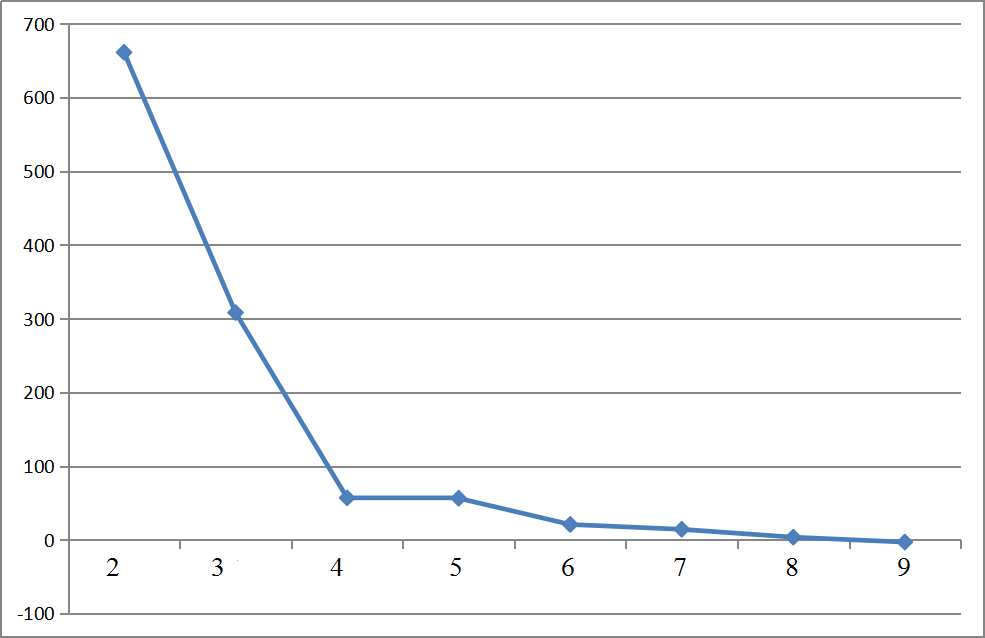

Supplement: Supplementary file 7 — Figure S2. ΔK values for the Structure analysis. (DOCX 34 kb) [file 12870_2019_1881_MOESM7_ESM.docx]
